# Combining Nitrous Oxide with Carbon Dioxide Decreases the Time to Loss of Consciousness during Euthanasia in Mice — Refinement of Animal Welfare?

**DOI:** 10.1371/journal.pone.0032290

**Published:** 2012-03-15

**Authors:** Aurelie A. Thomas, Paul A. Flecknell, Huw D. R. Golledge

**Affiliations:** 1 Comparative Biology Centre, Medical School, Newcastle University, Newcastle Upon Tyne, United Kingdom; 2 Institute of Neuroscience, Medical School, Newcastle University, Newcastle Upon Tyne, United Kingdom; Université Pierre et Marie Curie, France

## Abstract

Carbon dioxide (CO_2_) is the most commonly used euthanasia agent for rodents despite potentially causing pain and distress. Nitrous oxide is used in man to speed induction of anaesthesia with volatile anaesthetics, via a mechanism referred to as the “second gas” effect. We therefore evaluated the addition of Nitrous Oxide (N_2_O) to a rising CO_2_ concentration could be used as a welfare refinement of the euthanasia process in mice, by shortening the duration of conscious exposure to CO2. Firstly, to assess the effect of N_2_O on the induction of anaesthesia in mice, 12 female C57Bl/6 mice were anaesthetized in a crossover protocol with the following combinations: Isoflurane (5%)+O_2_ (95%); Isoflurane (5%)+N_2_O (75%)+O_2_ (25%) and N_2_O (75%)+O_2_ (25%) with a total flow rate of 3l/min (into a 7l induction chamber). The addition of N_2_O to isoflurane reduced the time to loss of the righting reflex by 17.6%. Secondly, 18 C57Bl/6 and 18 CD1 mice were individually euthanized by gradually filling the induction chamber with either: CO_2_ (20% of the chamber volume.min−1); CO_2_+N_2_O (20 and 60% of the chamber volume.min^−1^ respectively); or CO_2_+Nitrogen (N_2_) (20 and 60% of the chamber volume.min−1). Arterial partial pressure (P_a_) of O_2_ and CO_2_ were measured as well as blood pH and lactate. When compared to the gradually rising CO_2_ euthanasia, addition of a high concentration of N_2_O to CO_2_ lowered the time to loss of righting reflex by 10.3% (P<0.001), lead to a lower P_a_O_2_ (12.55±3.67 mmHg, P<0.001), a higher lactataemia (4.64±1.04 mmol.l^−1^, P = 0.026), without any behaviour indicative of distress. Nitrous oxide reduces the time of conscious exposure to gradually rising CO_2_ during euthanasia and hence may reduce the duration of any stress or distress to which mice are exposed during euthanasia.

## Introduction

Many millions of mice are used worldwide for scientific purposes, for example; approximately 3 million mice are currently used per year in the United Kingdom for scientific research [1]. Virtually all of these mice are euthanized at the end of the study. Since carbon dioxide (CO_2_) is the most common method of euthanasia in laboratory rodents [2]–[5], the majority of these mice will be killed using this agent. Carbon dioxide is an anaesthetic agent; its minimum alveolar concentration (MAC) is 403 mmHg (approximately 50% atm) in rats [6]. Carbon dioxide has been shown to dose-dependently acidify cerebrospinal fluid pH, consequently inhibiting central N-Methyl-D-Aspartate (NMDA) receptors [7], which may partially explain its mechanism of anaesthetic action.

For ethical and legal reasons (e.g. European directive 2010/63EU, and the Animals (Scientific Procedures) Act(1986) in the United Kingdom) it is essential to ensure that the process of euthanasia is carried out with as little pain and distress as possible. Despite the popularity of CO_2_ for euthanasia, it has been shown that exposure to CO_2_ is aversive to rodents [8]–[10]. Exposure to high concentrations of CO_2_ may lead to pain in rodents, since concentrations above 37% cause activation of nociceptive pathways in rats [11]–[12] and exposure of humans to similar concentrations is reported as painful [3]. Because loss of consciousness is expected to occur when the inspired concentration of CO_2_ is approximately 30%, the UK recommendations for good practice of CO_2_ euthanasia suggest the use of a gradually rising concentration of CO_2_. The use of 100% CO_2_ at a flow rate of 20% of the Chamber Volume per Minute (CV.min^−1^) has been shown to produce loss of consciousness without evidence of pain, but not without the potential of rats experiencing stress or distress induced by mechanisms other than nocicpetion [13]. Rodents show behavioural aversion to CO_2_ at levels substantially below those expected to activate nociceptive pathways [11]–[12]. There are several potential mechanisms, which may underlie this aversion. Exposure to low concentrations of CO_2_ (approximately 7%) causes dyspnoea in humans, which becomes severe at around 15% [14]. The possibility that conscious rodents may be experiencing dyspnea when exposed to CO_2_ is a potential welfare problem, as it is known that dyspnoea can be highly distressing in humans [15]–[17] and shares many features with pain [18]–[19]. It is possible that rodents may experience similar levels of dyspnoea at the same concentrations, although this is difficult to detect, as, like pain, dyspnoea is a subjective experience. In addition, inhaled CO_2_ has been shown to evoke fear behavior via activation of limbic structures including the amygdala in mice [20].

Given the evidence of rodent aversion to CO_2_ and the range of potential mechanisms which may induce fear and distress it would be desirable to shorten the duration of exposure to CO_2_ during euthanasia procedures if this could be achieved without either increasing the concentration of CO_2_ to which animals are exposed or increasing the level of stress and/or distress which animals experience. One potential method for shortening CO_2_ exposure duration is the addition of Nitrous Oxide to the euthanasia gas mixture. Nitrous Oxide (N_2_O) is a low solubility anaesthetic agent with mild analgesic properties [21]–[22]. The MAC of N_2_O lies within the range 150% and 235% in rats [23]–[25]. Inhaling high concentrations of N_2_O in combination with a volatile anaesthetic agent lowers the time to induction of anaesthesia in many species [26]–[29] via a mechanism commonly referred to as the “second gas effect” [30]. Kitahata and colleagues (1971) demonstrated that this second gas effect also occurred when N_2_O was combined with CO_2_ in cats. To our knowledge this effect has never been demonstrated in rodents [31].

This study assessed whether the addition of a high inspired concentration of N_2_O to a standard gradually rising concentration of CO_2_ (as is typically used for rodent euthanasia) would lower the time to loss of consciousness. A pilot study also examined whether combination of N_2_O with Isoflurane reduced the time to loss of consciousness in mice. In both cases, the existence of a second-gas effect would shorten the conscious experience of any potential distress experienced by mice during the euthanasia process and represent a potential animal welfare refinement if used in routine practice.

## Results

### Pilot study

The mice lost their righting reflex after 91.0±11.0 s when isoflurane was carried in 100% oxygen. The righting reflex was lost significantly faster when isoflurane was carried in 25% O_2_ and 75% N_2_O (69.7±7.0 s, P<0.0001, t-test). None of the mice receiving N_2_O alone (treatment 3) lost their righting reflex. Some ataxia was noted in this group as well as intermittent periods of immobility, but there were no obvious signs indicating distress or aversion.

### Main study

#### Time to loss of righting reflex

**Figure 1:**
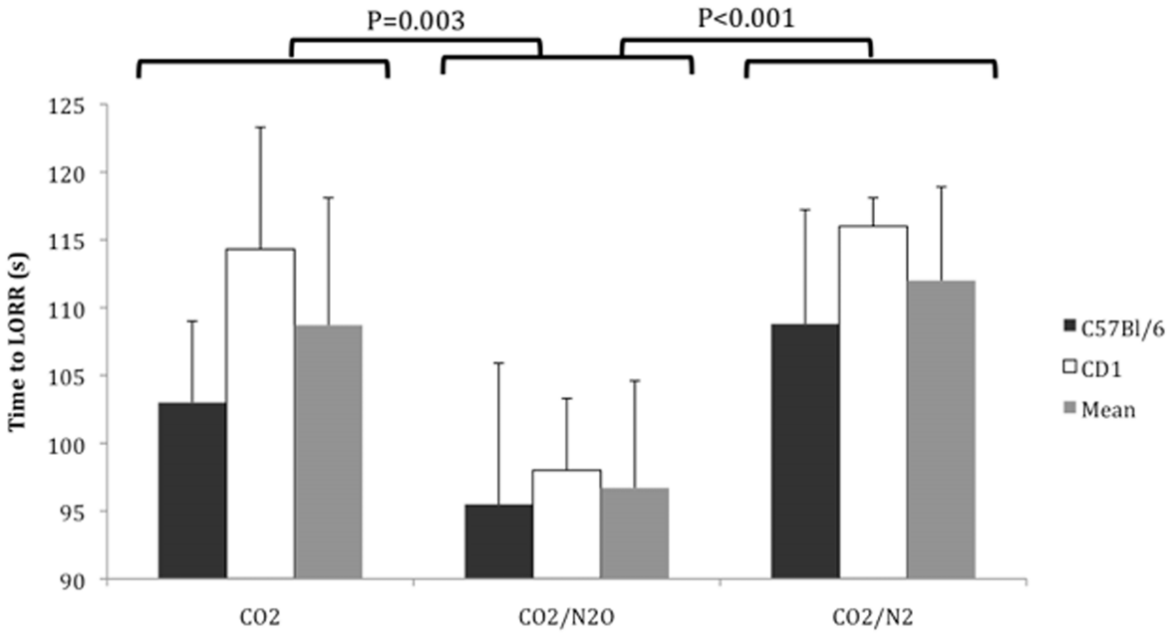
Time to loss of righting reflex (LORR) in seconds within the 3 treatment groups and the 2 strains of mice (C57Bl/6 and CD1). Error bars: +1 SD. Composition of the gas mixtures for the groups 1–3: see table 3.

The anaesthetic agent significantly affected the time to loss of the righting reflex (see Figure 1, P<0.001, 3-way ANOVA) Mice anaesthetized with the mixture of CO_2_ and N_2_O reached LORR significantly faster (96.7±7.9 s) than those anaesthetized with CO_2_ alone (108.7±9.4 s, P = 0.003) or CO_2_ in N_2_ (112.4±6.9 s, P<0.001). There was also a significant main effect of strain upon time to loss of consciousness, for all treatment conditions C57BL/6 mice lost consciousness faster than CD1 mice (P = 0.009). There was no significant effect of the sex of the mice and no significant interactions between factors. For CO_2_ group, the mean time to LORR corresponds to a F_i_CO_2_ of 24% at LORR; against 20% for the CO_2_/N_2_O and 27% for the CO_2_/N_2_ group.

#### Blood gas analysis

Arterial blood from the left cardiac ventricle was successfully obtained from 28 mice (9 mice from group 1, 11 mice from group 2, 9 mice from group 3). For all samples, the P_a_CO_2_ was higher than the reportable range (>130 mmHg) of our blood gas analyzer. In one of the 11 blood samples obtained from the group 2 animals, no pH value was obtained. Average values for arterial blood pH, P_a_O_2_ and lactate are presented in table 1.

**Table 1:** Values of pH, P_a_O_2_ (mmHg) and lactate (mmol.l^−1^) measured in arterial blood at the time of loss of righting reflex.

Group	pH	PaO_2_	Lactate
1 – CO_2_	6.62±0.17 (n = 9)	30.0±9.90 (n = 9)	3.16±0.65 (n = 9)
2 – CO_2_/N_2_0	6.94±0.04 (n = 10)	12.55±3.67 (n = 11)	4.64±1.04 (n = 11)
3 – CO_2_/N_2_	6.89±0.04 (n = 9)	13.11±5.32 (n = 9)	5.92±1.67 (n = 9)

Composition of the gas mixtures for the groups 1–3: see table 3.

The pH was below 7.0 in all mice. The mice receiving CO_2_ alone for euthanasia were significantly more acidaemic (P<0.001, 3-way ANOVA) compared to the 2 other groups (P<0.001 for both cases). The pH was not significantly different between mice receiving Nitrogen or Nitrous Oxide, nor did pH differ significantly between strains or sexes.

PaO_2_ varied significantly between treatment groups (P<0.001). PaO_2_ was significantly lower when N_2_O or N_2_ were added to CO_2_ (P<0.001 for both comparisons). There were no significant main effects of strain or sex, but there was a significant interaction between strain and sex (P = 0.017). PaO_2_ did not differ significantly between the CO_2_+N_2_ and CO_2_+N_2_O groups.

Blood lactate varied significantly between treatment groups (P<0.001, three-way ANOVA). Lactate was significantly higher in the CO_2_+N_2_ and CO2+N_2_ groups than in the CO_2_ alone group (P = 0.026 and P<0.001 respectively). Lactate level did not differ significantly between the CO_2_+N_2_ and CO_2_+N_2_O groups.

#### Behaviour analysis

There was no significant difference between treatment groups with regards to the behaviours analyzed during the acclimatization period.

None of the mice jumped during their acclimatization period. The mice jumped when exposed to CO_2_ (0.8±1.4 jump.min^−1^) and significantly more often in the CO_2_/N_2_ group (2.6±3.9 jump.min^−1^, P = 0.039, 3-way ANOVA). None of the mice, in either strain, jumped when CO_2_ was combined to N_2_O.

The incidence of rearing was 4.6±3.4, 5.7±3.7 and 5.0±2.9 rears per minute during the acclimatization period preceding euthanasia with CO_2_, CO_2_/N_2_O and CO_2_/N_2_ respectively (P>0.05, 3-way ANOVA). Once the gases were delivered to the chamber, the rate of rearing decreased. The mice receiving CO_2_ with N_2_O reared significantly less once the gas flow started (1.29±1.36, P<0.001).

A strain effect was detectable for rearing and jumping: the C57Bl/6 mice reared and jumped more frequently than the CD1 mice (P<0.001 and P = 0.003 respectively). There was no significant effect of sex on the incidence of rearing.

## Discussion

Nitrous oxide has been used since the 19^th^ century as an analgesic. It has also been used to induce anaesthesia although its use as a sole anaesthetic in humans has declined because concentrations sufficient to induce even moderate levels of anaesthesia render patients hypoxic [32]. Currently, N_2_O is used mixed with O_2_ and general anaesthetic agents in human anaesthesia to lower the time to loss of consciousness [26], [33] and for its analgesic properties, traditionally for labour and dental pain relief [34]–[35]. In dogs, the inhalation of high concentrations of N_2_O concurrently with halothane was shown to cause accelerated uptake of the latter agent [30], [36]. Although this phenomenon was expected to speed induction of anaesthesia, N_2_O failed to improve the rate or quality of induction in dogs receiving newer inhalant agents (isoflurane and sevoflurane) when delivered in a 2∶1 N_2_O and O_2_ mixture [37]. There appear to be no published data describing the use of N_2_O to lower the time to induction of anaesthesia in rodents. We have shown that addition of N_2_O to both carbon dioxide and isoflurane reduces the time to loss of consciousness in mice. Nitrous oxide lowered the time to induction by 17% when inhaled concurrently with isoflurane (pilot study) and by 10% when combined with CO_2_ (main study).

The difference in magnitude of effect between the two agents could be explained by a number of mechanisms including a difference in the quantity of N_2_O inhaled by animals in the two studies, the difference in anaesthetic potency between CO_2_ and isoflurane, the strain effect noted in the main study or the difference of amplitude of cardiovascular side-effects caused by CO_2_ and isoflurane.

In our pilot study, N_2_O was delivered with a flow rate of 3 l.min^−1^ in each of the 3 groups. Our induction chamber had a volume of 7 litres, hence this represents a flow rate of 43% of the chamber volume per minute, in the main study 60% of the chamber volume of N_2_O was delivered. The mechanism explaining the “second gas effect” [30] relies on the rapid intake of a large volume of the very insoluble N_2_O from the lungs. Hence it is likely that the difference in volume of N_2_O delivered per minute had an impact on the speed of induction of anaesthesia.However, less N_2_O was delivered in combination with Isoflurane, yet the time to induction was shorter. If the difference in inspired fraction (F_i_) of N_2_O was the main factor responsible for the difference of amplitude, we would then have expected the mice from our pilot study to have a delayed induction compared to the mice from the main study. It is therefore unlikely that the difference of F_i_ of N_2_O could explain the difference in time to LORR between our pilot and our main study.

Bispectral Index (BIS) is a neurophysiological monitoring technique which has been shown to correlate reliably with depth of anaesthesia in humans, Peyton and collaborators (2008) showed that the BIS was significantly lower (i.e. the anaesthesia was deeper) when the patients were induced with sevoflurane in 30% O_2_ and 70% N_2_O than with sevoflurane in 100% O_2_ [38]. Given that N_2_O itself does not alter the bispectral index [39] Peyton *et al*'s results suggest that the second gas effect is more likely to be due to a higher early arterial partial pressure of anaesthetic agent rather than a sub-anaesthetic action of the N_2_O *per se*. In this regard, it is very likely that the difference in potency between CO_2_ (MAC = 50% in rats; [6]) and isoflurane (MAC = 1.41% in mice; [40]–[41]) explains at least partially the difference of magnitude of effect in the two studies. Beyond the absolute potency of isoflurane and CO_2_ as anaesthetic agents, the relative dose administered to our mice was different in the pilot and the main study. After 1 min of gas supply, the mice in the pilot study were receiving the equivalent of 1.52xMAC Isoflurane (F_i_ = 5%×43% CV.min^−1^, MAC = 1.41%) whereas the mice in the main study received only 0.4xMAC CO_2_ (F_i_ = 20% CV.min^−1^, MAC = 50%). This difference of dose received by the mice during their first minute of gas exposure could be part of the explanation for the smaller (but still statistically significant) amplitude of the N_2_O effect in our main study.A significant strain difference was consistently present (time to LORR, blood gas changes as well as behavioural analysis). In the case of time to LORR the C57Bl/6 mice lost consciousness faster than the CD1 mice. It is possible that we introduced some bias by using 2 different strains for our main study but only one, C57Bl/6 for our pilot study. Mouse strain might modestly influence MAC for isoflurane. Sonner and collaborators (1999) however showed that C57Bl/6 and CD1 mice have very similar MAC for isoflurane (1.30 and 1.34% respectively) [41]. No data are currently available regarding the strain effect on CO_2_ MAC in mice. However it seems unlikely that the strain difference explains the difference of magnitude of effect between our pilot and main study.

Because the uptake of N_2_O is perfusion limited [42], its uptake will be optimal in lung compartments with a lower ventilation/perfusion (V/Q) ratio [30]. These compartments receive most of the blood flow and predominantly determine the composition of gases in arterial blood. The V/Q mismatch is a dynamic ratio during anaesthesia, depending on muscle relaxation, but also on the respiratory and cardio-vascular side-effects of the anaesthetic agent used. Isoflurane and N_2_O produce only moderate respiratory and cardiovascular depression in rodents [40] and are unlikely to significantly disrupt the V/Q ratio of lung compartments. Carbon dioxide on the other hand is known to have dramatic cardiovascular side-effects and to disrupt the V/Q ratio. At high concentration CO_2_ stimulates the nasal mucosa in rats and produces apnoea, as well as a strong and immediate vagally mediated bradycardia and a sympathetically mediated systemic and pulmonary hypertension [43]–[45]. These strong side-effects preclude the use of CO_2_ as an anaesthetic for anything other than very brief procedures [46]–[47]. It is likely that the severe bradycardia combined with the changes in pulmonary perfusion produced by CO_2_ would disrupt the V/Q ratio enough to alter N_2_O uptake and decrease the second gas effect, probably explaining why the mice lost consciousness only 10% faster when N_2_O was added to CO_2_ – compared to 17% faster when N_2_O was added to isoflurane.

The addition of nitrous oxide to carbon dioxide had a significant effect in reducing the time between first exposure to the gas and loss of consciousness. The presence of a high concentration of N_2_O in the gas mixture also accompanied a reduction in F_i_CO_2_ at the time of loss of righting reflex (F_i_CO_2_ = 24% in the CO_2_ group, compared to 20% in the CO_2_+N_2_O group). Given that the noxious threshold for CO_2_ was above 37% in rats [11]–[12], our study would support the view that the use of a gradually rising concentration of CO_2_ in the induction chamber allows the mice to lose consciousness before experiencing pain [13]. If higher CO_2_ flow rates are used, where noxious concentrations of CO_2_ would be reached before loss of consciousness, the addition of N_2_O could avoid the perception of CO_2-_induced pain. The benefit of the addition of high concentrations of N_2_O to CO_2_ at 20% chamber volume per minute CO_2_ flow rates however would primarily be in shortening the conscious exposure to the gas and therefore minimizing the duration of any period of distress. Since concentrations of CO_2_ below those likely to cause pain have been shown to produce aversion in both rats [9] and mice [10], it is reasonable to suggest that N_2_O may reduce any period of such distress during the procedure. Whether a 10% reduction in this period represents a significant welfare benefit depends upon the degree and the type of distress experienced by mice during the euthanasia process.

The addition of N_2_O to the gas mixture had the expected physiological effects on the blood gas analyses. The mice were more severely hypoxic in the CO_2_/N_2_O and CO_2_/N_2_ groups than in the group 1, which underwent standard gradual-fill CO_2_ euthanasia. This was expected as the O_2_ initially present in the induction chamber would be more quickly displaced by the presence of a second gas in the euthanasia mixture. Despite the commonly held notion that hypoxia does not produce dyspnoea hypoxia was shown to generate an equivalent level of air hunger as hypercapnia in humans [48]–[49]. The ventilatory response to hypoxia furthermore depends on the level of CO_2_ [50] and it has been shown that in conscious humans the effect of progressive hypoxia on breathing is much greater if there is a concomitant increase in inspired CO_2_ [51]–[52]. In our study, N_2_O reduced the time spent conscious in the induction chamber. However, N_2_O also rendered the mice more severely hypoxic, and we cannot exclude the possibility that this may induce more severe air-hunger than induction with CO_2_ alone.

Studies carried out in people show that dyspnoea/air hunger is more than just a physical sensation and has an important affective component [53]–[54]). This affective component to the dyspnoeic sensation leads to distress in people [16]. Interestingly, recent studies indicate that the perception of dyspnoea and pain involves similar CNS structures [17], [19]. We attempted in our study to assess the potential distress experienced by the mice by scoring behaviours potentially associated with distress.

Amongst the four behavioural events analyzed, the incidence of jumping is the most interesting. Jumping involved an intense springing upwards of the mouse with a sudden extension of the hindlimbs. The amplitude of the jump was such that the mice frequently collided with the roof of the induction chamber (14 cm height). Jumps frequently occurred in rapid succession (3–6 jumps), in-between each bout of jumping the animals appeared normally conscious and ambulatory. None of the animals exhibited any jumping behaviour during the acclimatisation period, reinforcing the authors' belief that these jumps are not part of the normal behavioural repertoire of this species. Irwin (1968) described “popcorn” jumping behaviours in mice in his behavioural and physiologic state assessment of the mouse [55]. Irwin describes this as “a seizure where the animal repeatedly “pops” into the air”. Although this description would fit our observation of the jumps, we consider it most unlikely that the animals were undergoing seizures since they appeared fully conscious immediately before and after jumps.

In this study, the mice exhibited jumping behaviour when exposed to CO_2_ at the recommended flow rate of 20% CV.min−1 and significantly more often when a high concentration of N_2_ was added to CO_2_ We interpret jumping as escape behaviour, possibly related to hypoxia. None of the mice exhibited jumping when N_2_O was added to CO_2_. This could be explained by a more rapid onset of sedation resulting from the combination of the two anaesthetic agents. Whereas unconsciousness is due to anaesthetic action into the brain, recent evidence suggests that immobilization under general anaesthesia is achieved by anaesthetic effect on the spinal cord [56]–[57]. Amongst the potential spinal receptors numerous results suggest the potential importance of NMDA receptors as mediators of the immobilizing capacity of inhaled anaesthetics [41], [58]. Both CO_2_ and N_2_O are known to act on central NMDA receptors, hence the combination of CO_2_ and N_2_O may enhance some spinal NMDA antagonism, leading to decreased mobility of the animal.

The mice from all three groups were acidaemic [59] and hypoxemic [60] at the time of loss of righting reflex. The hypoxemia is likely be explained by the low inspired O_2_ within all treatments. This was significantly more marked in groups 2 and 3, where N_2_ or N_2_O were administered along with CO_2_. The hypoxemia would have triggered an increase in anaerobic metabolism, producing lactic acid. Although in all groups the acidaemia is likely to be mainly of respiratory origin, the lactic component might be significant for the mice euthanized with CO_2_ and N_2_O or CO_2_ and N_2_ [61].

Whilst the level of CO_2_ to which mice are exposed during euthanasia is unlikely to cause pain by the direct action of CO_2_ upon mucosal surfaces [12] we cannot exclude the possibility that systemic lactic acidosis and consequent activation of ASIC channels could cause pain [62]–[67]. Were this to be the case then the increased lactate levels we observed when N_2_O was added could represent a welfare issue. However, we observed no behavioural evidence of this effect but further investigation would be required to confirm that Nitrous Oxide induced lactic acidosis is not detrimental to the welfare of mice killed with an N_2_O/CO_2_ mixture.

In conclusion, our main results suggest that the addition of a high concentration of N_2_O to a commonly recommended flow-rate of CO_2_ for the euthanasia of mice shortens the time to loss of consciousness by 10% without triggering any obvious increase in behavioural signs of aversion or distress. Therefore, nitrous oxide reduces the duration of conscious exposure to CO_2_ and potentially reduces the duration of distress. Determining whether N_2_O represents a worthwhile refinement to CO_2_ euthanasia in rodents requires further studies in order to accurately assess distress in mice during gradually raising CO_2_ exposure as well as the welfare impact on conscious rodents of severe hypoxemia and acidosis. Future studies should also examine what concentration of nitrous oxide is optimal for minimizing the duration of conscious exposure to CO_2_.

## Materials and Methods

### Ethical Statement

This study was carried out in accordance with project and personal licenses granted under the United Kingdom's Animals (Scientific Procedures) Act (1986) and Newcastle University Ethical Review Committee specifically approved this study at (PPL 60/4126 and 60/4058).

### Animals

A total of 18 CD1 mice (9 females, 9 males, aged 10–12 weeks, weight 26.9±0.5 g) and 30 C57Bl/6 mice (21 females, 9 males, aged 10–12 weeks, weight 25.6±0.7 g) were used in the study (Charles River, Kent, UK). All mice were housed in groups of 3 in individually ventilated cages (Arrowmight, Hereford, UK). The animal room was maintained at 23±1°C, 35% humidity and on a 12/12 h light/dark cycle (lights on at 07:00) with 15–20 air changes per hour. Food (CRM (P), SDS Ltd, Essex UK) and tap water were provided *ad libitum*. Sawdust bedding (Aspen, BS and S Ltd, Edinburgh, UK) was provided along with nesting material (Shredded paper, DBM, Broxburn, UK). All mice were allowed a period of at least 14 days of acclimatization before the procedure. Mice were maintained in a facility that was shown by serological monitoring to be free from all recognized rodent respiratory pathogens.

### Equipment

A 7-litre clear Plexiglas induction chamber (VetTech Solutions LTD, Congleton, UK) was connected to an anaesthesia machine (Selectatec SM, Cyprane Limited, Keighley, UK) equipped with CO_2_, N_2_O and N_2_ cylinders (B.O.C., Chester-Le-Street, UK). The gas mixture was delivered to the chamber via a single fresh gas inlet tube located underneath a multi-perforated plastic floor. The supply pipe had several outlet ports to ensure gas was supplied to the full length of the chamber. In order to optimize the blending of the mixture, 4 miniature fans were installed alongside the fresh gas inlet, under the perforated floor. These fans ensured that there was a linear increase in the concentration of each gas during filling and that gas concentrations and ratios were the same throughout the cage despite the different densities of the various gases.

**Figure 2:**
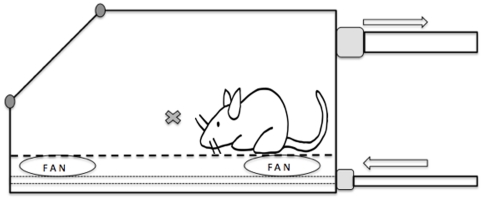
Diagram representing the induction chamber. The fresh gas inlet (bottom arrow) of the chosen mixture was delivered underneath a multi-perforated floor. A total of 4 mini fans were ensuring that the mixture was optimally blended. The sampling end of the sidestream gas monitoring (**X**) was positioned at mouse level, 8 cm above the floor. The gas outlet (upper arrow) was connected to an active scavenging system.

The scavenging port, located at the top of the chamber, was connected to an active scavenging system (Figure 2). The concentrations of the agents in the chamber (CO_2_, O_2_, N_2_O and Isoflurane) were monitored with a sidestream anaesthesia gas analyzer (VitaLogik 4500, Charter Kontron, Milton Keynes, UK). The end of the sampling tube of the gas analyzer was positioned 4 cm above the floor of the induction chamber in order to be at the approximate head level of a mouse walking in the chamber. The sampling rate of the gas analyzer was 50 ml/min. Once the F_i_CO_2_ reached the upper limit detectable by the anaesthesia monitor (13% atm), an indirect and retrospective calculation using F_i_O_2_ and F_i_N_2_O was used to calculate the CO_2_ concentration. When CO2 was delivered alone: [CO_2_] = 21-[O2]/0.21. When CO2 was delivered with N2O: [CO2] = A similar calculation was used to calculate F_i_N_2_.The induction chamber and the screen of the gas monitor were continually filmed by 2 high definition video cameras (HDR XR155E, Sony, Stuttgart, Germany) from the start of the procedure to the loss of consciousness of the mice.

Prior to the start of each procedure, the empty chamber was filled 3 times with the different gas mixtures and the linearity of the filling rate for each the gases was confirmed by linear regression.

### Pilot study

To the best of the authors' knowledge, there was no published data describing the use of N_2_O in mice and its potential effect on time to induction of anaesthesia. A pilot study was therefore conducted to assess whether nitrous oxide had demonstrable effects that could be associated with a “second gas effect” in mice.

A total of 12 C57Bl/6 female mice were used in a crossover study design. Each of the 12 mice received each of the 3 treatments (Table 2) with at least a 48 h washout period between treatments. The mice were individually placed in the induction chamber described above and were allowed a 2 minute acclimatization period after which the gases were delivered to the chamber. The loss of righting reflex (LORR) was confirmed by tilting of the chamber and the time to LORR was recorded as an indicator to loss of consciousness. LORR was taken as the point at which a recumbent mouse showed no attempt to right itself when the cage was tipped to a 45° angle. Once LORR was lost, gas inflow was discontinued, and the mice were removed and placed in an incubator at 30°C for 30 min before being returned to their home cage.

**Table 2:** Composition and flow rate of the anaesthetic mixtures used for anaesthesia induction – pilot study.

Treatment	Isoflurane (%)	O_2_ (%)	N_2_O (%)	Flow (l.min^−1^)
1	5	100	0	3
2	5	25	75	3
3	0	25	75	3

The Isoflurane % was read on the dial of the precision vaporizer, the O2 and N2O % referred to the Fi of the gases.

The time to loss of righting reflex was analyzed by repeated measures ANOVA (SPSS for Windows, Version 18, IBM Corporation, Sommer, NY, USA), with α = 0.005.

### Main study

A sample size calculation was made using the variance obtained in the pilot study (NQuery 7.0, Statistical Solutions, Boston, MA, USA). In order for the main study to reach 90% power and detect a difference in means at the 0.05 level the sample size was calculated at n = 12 mice per group. Twelve mice were subsequently allocated to one of 3 gas mixtures (Table 3) according to a randomized-block study design. Each of the 3 groups included 6 CD1 mice (3 males, 3 females), and 6 C57Bl/6 mice (3 males, 3 females).

**Table 3:** Composition and flow rates of the gas mixtures used for mice euthanasia- main study.

Group	n	CO_2_ (%CV.min^−1^)	N_2_O (%CV.min^−1^)	N_2_ (%CV.min^−1^)	Total (%CV.min^−1^)
1	12	20	/	/	20
2	12	20	60	/	80
3	12	20	/	60	80
**Total**	**36**				

CV.min−1: Chamber Volume per min. The induction chamber had a volume of 7 liters: 20% CV.min−1 = 1.4 l.min−1, 60% CV.min−1 = 4.2 l.min−1.

Each of the 3 gas mixtures supplied CO_2_ at a gradually rising concentration, with a flow rate of 20% CV.min^−1^ (1.4 l.min^−1^), which was equivalent to a commonly recommended CO_2_ flow rate (*e.g.*Hawkins *et al*, 2006). In group 1, no other gas was added to the CO_2_. In group 2, N_2_O at a rate corresponding to 60% CV.min^−1^ (4.2 l.min^−1^) was added to the CO_2_. In the 3^rd^ group, an inert gas, nitrogen (N_2_) at a rate corresponding to 60% of the CV.min^−1^ (4.2 l.min^−1^) was added to the CO_2_ in place of the N_2_O.

The mice were individually placed in the euthanasia chamber and the video recording was commenced. After 2 minutes of acclimatization the gas mixture 1, 2 or 3 was delivered to the chamber. The time to loss of righting reflex (LORR) was measured (sec) as an indicator for the loss of consciousness. The LORR was considered to have occurred when the mouse did not attempt to regain sternal recumbency after being rolled onto its back by tilting of the induction chamber. The video recording was stopped at this point.

The mouse was immediately taken out of the chamber and placed on a tightly fitted face-mask. The same gas mixture being supplied to the chamber was supplied via the face-mask. After ensuring that the mouse was unconscious, a cardiac puncture was performed by an experienced operator. The left ventricular blood sample obtained was immediately analyzed and the following parameters were measured: pH, arterial partial pressure of oxygen (P_a_O_2_) and CO_2_ (P_a_CO_2_) in mmHg, and lactate in mmol/l (iStat, Abbott, Princeton, NJ, USA). Death was ensured by cervical dislocation of the unconscious animal.

The video-recordings were retrospectively analyzed by a single observer in a blind manner using the behavioural scoring software Observer® XT (Noldus, Wageningen, Netherlands). Rearing, and jumping were codified using the software. Rearing was defined as a bipedal posture, the mouse standing on its hind limbs, with or without placement of the fore limbs onto the wall of the box. Jumping was defined as a sudden springing off the ground where all four feet left the cage floor. The incidence of these behaviours was quantified during the acclimatization period and during the euthanasia gas exposure period of each animal.

The F_i_CO_2_ (%) at time for LORR was retrospectively calculated based on the mean time to LORR for each of the 3 groups and linear regression of the F_i_CO_2_ based on the preliminary filling rate of the empty induction chamber.

### Statistical analysis

The data are presented as mean ± standard deviation (SD). Statistical analyses were carried out using SPSS for Windows (Version 19.0, IBM Corporation, Sommer, NY, USA). The effect of treatment, strain and sex upon time to loss of righting reflex (sec), pH, PaO_2_ (mmHg), lactate (mmol.l^−1^), and the incidence of 3 behavioural events (rearing, climbing, jumping) were analysed using three way analyses of variance. Post-hoc analyses were performed using Tukey's honestly significant difference. Data are presented as mean ± 1 S.D. Differences were considered significant at P<0.05. Where data did not differ significantly between sexes or strains, averages data for each treatment group are presented as the combined data for both strains and/or sexes.
